# Congestive Heart Failure Is Associated With Worse Outcomes in Patients With Ischemic Colitis: A Nationwide Study

**DOI:** 10.7759/cureus.24308

**Published:** 2022-04-20

**Authors:** Ratib Mahfouz, Landon A Kozai, Adham E Obeidat, Mohammad Darweesh, Mahmoud M Mansour, Mustafa F Douglas, Eric Berthiaume

**Affiliations:** 1 Internal Medicine, Kent Hospital/Brown University, Warwick, USA; 2 Internal medicine, University of Hawaii, Honolulu, USA; 3 Internal Medicine, University of Hawaii, Honolulu, USA; 4 Internal Medicine, East Tennessee State University, Johnson City, USA; 5 Internal Medicine, University of Missouri School of Medicine, Columbia, USA; 6 Internal Medicine, Midwestern University Arizona College of Osteopathic Medicine, Sierra Vista, USA; 7 Gastroenterology, Kent Hospital/Brown Unviersity, Warwick, USA

**Keywords:** bowel ischemia, colonic ischemia, colectomy, congestive heart failure, ischemic colitis

## Abstract

Introduction: Ischemic colitis (IC) results from compromised blood flow to the colon. Risk factors include atrial fibrillation (A.Fib), peripheral artery disease (PAD), coronary artery disease (CAD), and congestive heart failure (CHF). However, few studies compared the mortality rate and colectomy between patients with IC with CHF and IC alone.

Objective: We aim to investigate the possibility of worse outcomes in patients with IC and CHF compared to IC alone.

Methodology: Using the National Inpatient Sample database from 2016 to 2019, we obtained baseline demographic data, total hospital charge, rate of colectomy, length of hospital stay (LOS), and in-hospital mortality. Data were compared using a t-test and chi-squared. Odds ratios for comorbidities including A.Fib, CAD, PAD, end-stage renal disease, chronic obstructive pulmonary disease, hyperlipidemia, hypertension, diabetes, and cirrhosis were calculated.

Results: 106,705 patients with IC were identified, among which 15,220 patients also had CHF. IC patients with CHF had a longer LOS (6.6 days vs 4.4 days; P<0.0001), higher total hospital charge ($71,359 vs $45,176; P<0.0001), higher mortality rate (8.5% vs 2.9%; P<0.0001), and higher colectomy rate (9.2% vs 5.9%; P<0.0001).

Conclusion: CHF is associated with poor outcomes in patients with IC. Our study showed an increased risk of mortality and colectomy compared to patients with IC alone. The findings suggest it may be warranted to have a heightened clinical suspicion of IC in patients with CHF who present with bleeding per rectum.

## Introduction

Ischemic colitis (IC) is a form of bowel ischemia resulting from hypoperfusion to the colon and is relatively common in the United States (US). The incidence of IC has been steadily increasing over the past four decades [1]. Prior studies show that states of hypoperfusion such as congestive heart failure (CHF) or sepsis, atherosclerosis, and constipation are common risk factors for developing IC [2,3]. The clinical presentation of IC is dependent on several variables such as the acuity of the ischemic event, presence of vascular collaterals, duration of ischemia, and occurrence of reperfusion injury. The resulting colonocyte death is reflected by the gross presence of subepithelial hemorrhage, edema, mucosal ulceration, or irreversible outcomes such as fulminant colitis and gangrene [4]. Patients typically complain of crampy epigastric pain, hematochezia, and tenesmus [2]. IC is often underdiagnosed due to its nonspecific clinical presentation.

CHF is a common condition that results when the heart fails to sustain the body's metabolic demands. In this setting, the colon, which relies heavily on vascular collaterals to maintain its blood supply is susceptible to low perfusion ischemic injury. In addition, CHF causes reduced cardiac output and, therefore, may be a risk factor in determining the severity of IC.

Previous studies have concluded a higher risk of mortality and colectomy rates in patients with IC who also have significant comorbidities such as cirrhosis and chronic obstructive pulmonary disease (COPD) [5,6]. However, there are no studies that have examined the relationship between IC and CHF. Thus, this study will aim to investigate outcomes in hospitalized patients with IC and CHF compared to those with IC alone.

## Materials and methods

Data source

Our study is a retrospective cohort study of patients admitted to hospitals with a primary diagnosis of IC in the United States between the years 2016 and 2019. The data were extracted from the Healthcare Cost and Utilization Project National Inpatient Sample (NIS) database. The NIS is sponsored by the Agency for Healthcare Research Quality (AHRQ) and is considered the largest publicly available inpatient health care database in the United States. The database includes data from at least 46 states and covers more than 97% of the US population [7]. A 20% probability sample was collected and subsequently weighted to ensure that the selected population was nationally representative. Each admission in the database was assigned one principal diagnosis, up to 40 secondary diagnoses, and 25 procedures. These variables are defined via the International Classification of Disease, 10th revision, and Clinical Modification (ICD-10-CM) codes.

Study variables

Baseline demographic data, including age, gender, and race; hospital information, including region and region; comorbidities, including atrial fibrillation (A.Fib), coronary artery disease (CAD), peripheral arterial disease (PAD), end-stage renal disease (ESRD), chronic obstructive pulmonary disease (COPD), hyperlipidemia (HLD), hypertension (HTN), diabetes mellitus (DM), and cirrhosis; and outcome variables, including colectomy rate, in-hospital mortality rate, total hospital charge, and length of stay were collected. The data were compared using a t-test and chi-square.

Statistical analysis

The statistical analysis was performed using STATA software, version 17.0 (StataCorp., College Station, TX, USA). The characteristics of patients with OSA alone and those who had both OSA and GERD were described using descriptive statistics. In this study, multivariate logistic regression analyses were performed to determine factors associated with in-hospital mortality. Variables that were not statistically significant (p-value > 0.05) on univariate analysis were excluded from the multivariate analysis, except for PAD, as it is considered an important risk factor for IC. The odds ratio at 95% CI was used to describe the association between the study and outcome variables. Statistical significance was defined as a two-tailed p-value of <0.05.

## Results

Patient and hospital characteristics

**Figure 1:**
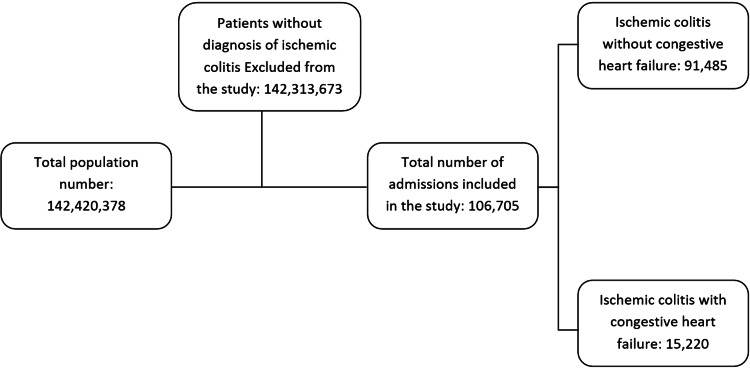
Inclusion and exclusion criteria for patients with ischemic colitis with or without congestive heart failure

We identified 106,705 patients with IC, among which 15,220 patients also had CHF diagnoses (Figure 1).

Patients with IC and CHF were older than patients with IC alone (mean of 75.2 vs. 69.6 years, P<0.0001). In comparison to patients without CHF, patients with IC with CHF had an increased prevalence of males (34% vs. 25%; p-value < 0.001) and black race (12% vs. 6.6%; p-value < 0.001). Most patients were predominantly white females in both groups. The differences between hospital characteristics and insurance type were statistically significant (Table 1).

**Table 1:** Demographics and clinical characteristics comparison between patients with IC and CHF compared to patients with IC alone IC: Ischemic colitis; CHF: Congestive heart failure; COPD: Chronic obstructive pulmonary disease; A.Fib: Atrial fibrillation; CAD: Coronary artery disease; PAD: Peripheral arterial disease; ESRD: End-stage renal disease; HLD: Hyperlipidemia; HTN: Hypertension; DM: Diabetes mellitus.

Characteristics	IC and CHF N = 15220 (14%)	IC without CHF N = 91485 (86%)	P-value
Age (years)	75.2	69.6	< 0.0001
Gender			< 0.0001
Males	5,158 (34%)	23,337 (25%)	
Females	10,061 (66%)	68,147 (75%)	
Race			< 0.0001
White	11,395 (75%)	73,197 (80%)	
Black	1,790 (12%)	6,074 (6.6%)	
Hispanic	965 (6%)	5,526 (6%)	
Others	1,070 (7%)	6,688 (7.4%)	
Hospital region			< 0.0001
Northeast	3,166 (20%)	19,413 (21%)	
Midwest	4,389 (29%)	22,460 (24%)	
South	5,135 (34%)	32,587 (36%)	
West	2,530 (17%)	17,025 (19%)	
Hospital bed size			< 0.0001
Small	2,890 (19%)	20,657 (23%)	
Medium	4,740 (31%)	28,845 (31%)	
Large	7,590 (50%)	41,982 (46%)	
Comorbidities			< 0.0001
COPD	4,720 (31%)	13,229 (14.4%)	
A. Fib	6,720 (44%)	11,436 (12.5%)	
CAD	8,850 (58%)	19,642 (21%)	
PAD	1,525 (10%)	4,647 (5.1%)	
ESRD	1,855 (12%)	2,360 (2.6%)	
Cirrhosis	475 (3.1%)	1,473 (1.6%)	
HLD	8,170 (54%)	44,087 (48%)	
HTN	13,700 (90%)	69,382 (76%)	
DM	6,551 (43%)	22,222 (24%)	

Inpatient outcomes

Mortality

**Figure 2:**
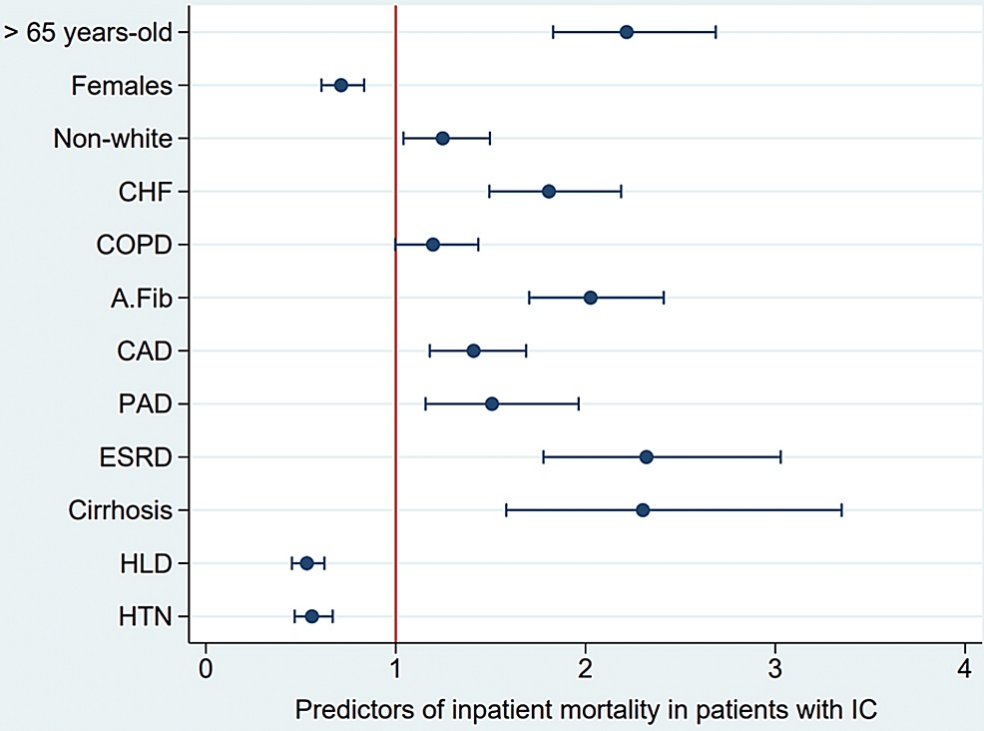
Odds ratio plot for predictors of mortality in ischemic colitis patients CHF: Congestive heart failure; COPD: Chronic obstructive pulmonary disease; A.Fib: Atrial fibrillation; CAD: Coronary artery disease; PAD: Peripheral arterial disease; ESRD: End-stage renal disease; HLD: Hyperlipidemia; HTN: Hypertension; DM: Diabetes mellitus.

The rate of death in IC patients with CHF was higher than in IC without CHF (8.5% vs. 2.9%, p-value < 0.001) (Table 2), which was also reflected in multivariate analysis with an odds ratio of 1.83 (p-value < 0.001) after adjusting for age, gender, race, and comorbidities (COPD, A.Fib, CAD, PAD, ESRD, cirrhosis, HLD, and HTN). Patients older than 65 years old had an odds ratio of 2.22 of dying during hospitalization (p-value < 0.001). Females had an odds ratio of 0.7 (p-value < 0.005). Non-white patients had an odds ratio of 1.27 (p-value 0.011). ESRD was the most statistically significant studied risk factor for in-hospital mortality (OR 2.44, 95% CI 1.86-3.2, p-value < 0.001), followed by cirrhosis (OR 2.34, 95% CI 1.6-3.41, p-value < 0.001). A.Fib, CHF, PAD, then CAD. HLD and HTN were associated with statistically significant decreases in the odds ratio of mortality (p-value < 0.001). COPD was associated with an increase in OR but was not statistically significant (p-value 0.051) (Table 3). A plot summarizes the results shown in Figure 2.

**Table 2:** Comparison of outcomes between patients with ischemic colitis and congestive heart failure vs ischemic colitis alone IC: Ischemic colitis; CHF: Congestive heart failure; USD: United States Dollar.

Outcome	IC + CHF	IC without CHF	P-value
LOS (Days)	6.6	4.4	< 0.0001
Colectomy	1,400 (9.2%)	5,196 (5.7%)	< 0.0001
Died	1,291 (8.5%)	2,644 (2.9%)	< 0.0001
Total charge (USD)	$71,359	$45,176	< 0.0001

 

**Table 3:** Odds ratio table for predictors of mortality in ischemic colitis patients CHF: Congestive heart failure; COPD: Chronic obstructive pulmonary disease; A.Fib: Atrial fibrillation; CAD: Coronary artery disease; PAD: Peripheral arterial disease; ESRD: End-stage renal disease; HLD: Hyperlipidemia; HTN: Hypertension; DM: Diabetes mellitus.

Mortality	Unadjusted OR (95% CI)	P-value	Adjusted OR (95% CI)	P-value
Age				
18-65 year	Reference		Reference	
>65 year	2.14 (1.79-2.56)	<0.001	2.22 (1.84-2.7)	<0.001
Gender				
Male	Reference		Reference	
Female	0.58 (0.5-0.67)	<0.001	0.7 (0.6-0.83)	<0.001
Race				
White	Reference		Reference	
Non-White	1.3 (1.1-1.53)	0.002	1.27 (1.07-1.52)	0.011
Comorbidities				
CHF	3.12 (2.68-3.63)	<0.001	1.83 (1.52-2.22)	<0.001
COPD	1.57 (1.33-1.86)	<0.001	1.2 (1.0-1.44)	0.051
A. Fib	3.01 (2.59-3.5)	<0.001	2.03 (1.7-2.4)	<0.001
CAD	2.02 (1.75-2.35)	<0.001	1.43 (1.2-1.71)	<0.001
PAD	2.8 (1.65-2.61)	<0.001	1.48 (1.13-1.92)	0.004
ESRD	3.5 (2.78-4.38)	<0.001	2.44 (1.86-3.2)	<0.001
Cirrhosis	2.83 (2-3.99)	<0.001	2.34 (1.6-3.41)	<0.001
HLD	0.6 (0.51-0.69)	<0.001	0.54 (0.46-0.63)	<0.001
DM	1.08 (0.93-1.27)	0.281		
HTN	0.77 (0.65-0.9)	0.001	0.57 (0.47-0.68)	<0.001

Length of Stay and Total Hospital Charges

In IC patients with CHF, the mean LOS was statistically significantly longer than in IC without CHF (6.6 vs. 4.4 days, p-value < 0.001). Total hospital charges were also significantly higher in the IC and CHF group ($71,359 vs. $45,176, p-value < 0.001). Table 2 summarizes these findings.

Colectomy

**Figure 3:**
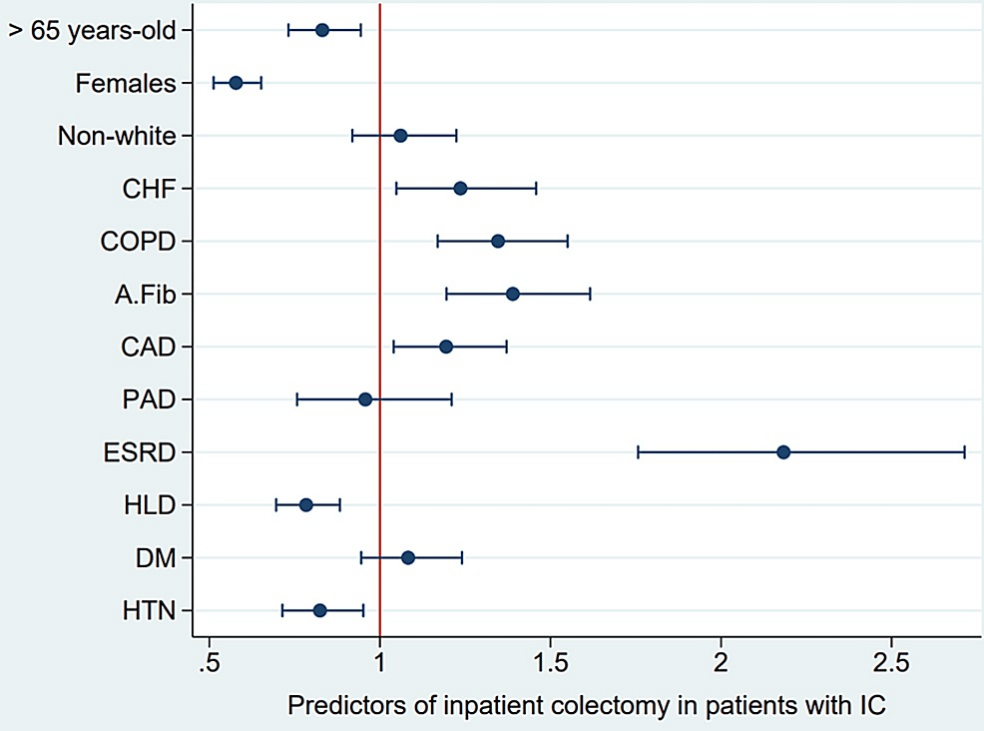
Odds ratio plot for predictors of colectomy in ischemic colitis patients CHF: Congestive heart failure; COPD: Chronic obstructive pulmonary disease; A.Fib: Atrial fibrillation; CAD: Coronary artery disease; PAD: Peripheral arterial disease; ESRD: End-stage renal disease; HLD: Hyperlipidemia; HTN: Hypertension; DM: Diabetes mellitus.

Patients with IC and CHF had an increased rate of in-hospital colectomy compared to patients with IC without CHF (9.2% vs. 5.7%, p-value < 0.001) (Table 2), which was also reflected in multivariate analysis with an increased odds ratio of 24% (p-value < 0.001) after adjusting for age, gender, race, and comorbidities (COPD, A.Fib, CAD, PAD, ESRD, HLD, DM, and HTN). Patients older than 65 years old and females had an odds ratio of 0.83 and 0.58 to undergo colectomy, respectively (p-value < 0.005). Non-white patients were slightly more likely to undergo colectomy but not statistically significant (p-value 0.41). ESRD was the most statistically significant studied risk factor for in-hospital colectomy (OR 2.19, 95% CI 1.8-2.72, p-value < 0.001), followed by A.Fib, COPD, CHF, then CAD. HLD and HTN were associated with a statistically significant decrease in the odds ratio of colectomy (p-value < 0.009). PAD and DM were not statistically significant (p-value > 0.05) (Table 4). A plot summarizes the results shown in Figure 3.

**Table 4:** Odds ratio table for predictors of colectomy in Ischemic colitis patients CHF: Congestive heart failure; COPD: Chronic obstructive pulmonary disease; A.Fib: Atrial fibrillation; CAD: Coronary artery disease; PAD: Peripheral arterial disease; ESRD: End-stage renal disease; HLD: Hyperlipidemia; HTN: Hypertension; DM: Diabetes mellitus.

Colectomy	Unadjusted OR (95% CI)	P-value	Adjusted OR (95% CI)	P-value
Age				
18-65 year	Reference		Reference	
>65 year	0.82 (0.73-0.92)	0.001	0.83 (0.73-0.94)	0.004
Gender				
Male	Reference		Reference	
Female	0.5 (0.45-0.57)	<0.001	0.58 (0.51-0.65)	<0.001
Race				
White	Reference		Reference	
Non-White	1.19 (1.03-1.36)	0.012	1.06 (0.92-1.22)	0.41
Comorbidities				
CHF	1.68 (1.46-1.93)	<0.001	1.24 (1.04-1.46)	0.011
COPD	1.45 (1.26-1.66)	<0.001	1.35 (1.17-1.55)	<0.001
A. Fib	1.59 (1.39-1.81)	<0.001	1.39 (1.2-1.61)	<0.001
CAD	1.43 (1.27-1.6)	<0.001	1.19 (1.04-1.37)	0.012
PAD	1.18 (0.94-1.47)	0.151	0.96 (0.78-1.21)	0.719
ESRD	2.96 (2.43-3.6)	<0.001	2.19 (1.8-2.72)	0.000
Cirrhosis	1.18 (0.8-1.73)	0.408		
HLD	0.78 (0.7-0.87)	<0.001	0.78 (0.69-0.88)	<0.001
DM	1.25 (1.1-1.4)	<0.001	1.08 (0.95-0.124)	0.241
HTN	0.85 (0.75-0.97)	0.015	0.82 (0.71-0.95)	0.008

## Discussion

This retrospective study is the first to evaluate the effect of CHF on outcomes in hospitalized patients with IC. Our results are concordant compared to similarly designed studies that examined COPD and cirrhosis instead of CHF [5,6]. Furthermore, the findings suggest that patients with IC will suffer worse outcomes, including higher mortality, colectomy rates, length of stay, and costs if carrying a co-diagnosis of CHF.

When adjusting for age, gender, race, and various comorbidities, we found that patients with IC and CHF had a nearly three-fold increase in mortality in patients with IC and coexisting CHF. Additionally, most of the other comorbidities included in the analysis, some of which are known risk factors for the development of IC, were associated with worse outcomes when combined with IC. This may reflect a reduced colonic vascular reserve in response to the hemodynamic derangements caused by CHF [8]. Outcomes are also worse with CHF in other vascular pathologies where perfusion is impaired, such as peripheral artery disease [9,10]. Consequences of CHF such as sympathetic vasoconstriction, reduced cardiac output, venous congestion, pulmonary edema are shown to have deleterious effects on the colon, which physiologically receives less blood flow compared to other parts of the gastrointestinal tract and has poor autoregulatory capability in response to hypotension [11-14].

Mortality in patients with IC may occur secondary to the complications of bowel necrosis, leading to sepsis and multiorgan failure, which in turn obligates one to undergo respective surgery. The adverse hemodynamic changes of CHF likely predispose the colon to such irreversible complications and, therefore, colectomy. We found that CHF increases the risk of undergoing colectomy in hospitalized patients with IC. Moreover, patients undergoing colectomy due to complications of IC face higher perioperative mortality with a co-diagnosis of CHF [15,16].

Although CHF per se is associated with high perioperative mortality in non-cardiac surgery [17], the degree of LV dysfunction, which was not included in our analysis, is associated with worse perioperative outcomes [18]. Furthermore, this analysis did not clarify the degree to which perioperative mortality comprised overall mortality. Given that IC is typically preceded by hypoperfusion, the degree of LV dysfunction may correlate with the severity of presentation in IC, although this has not been studied.

In keeping with higher mortality rates and colectomy, we observed that those with IC and CHF had a longer LOS and higher hospital charges. This was likely directly related to increased rates of colectomy and the need for additional medical treatments or surgical interventions. The type of surgery used to perform the colectomy was not specified, which could have affected the results. Laparoscopic colectomies were associated with reduced hospital LOS compared to open colectomies in patients with IBD [19]. Furthermore, the acuity of patients' CHF was not described. Acute CHF exacerbations compounded with IC could lead to increased costs and utilization of resources.

The population study was significantly skewed toward the female gender and slightly older adults. The older adults may have a lower physiologic reserve or other comorbidities unaccounted for by the study, resulting in worse outcomes. It remains unclear why the female gender or Black race was associated with worse outcomes. Perhaps this reflects inequities in healthcare delivery to specific populations. Greater age and male sex were independently associated with mortality in IC in a large population-based study [1], which was only partially consistent with our results.

This study has several limitations. The first is its retrospective nature. Specific details such as the severity of heart failure and specific heart failure syndrome (i.e., preserved or reduced ejection fraction) were not obtained. Additionally, the mortality rates associated with IC vary depending on the area of the colon involved, with right-sided IC and pan-colonic ischemia being worse than isolated left-sided IC [20-22]. Therefore, we did not include this data in the analysis, although it could have affected the outcome.

## Conclusions

In our study, the diagnosis of CHF and IC is associated with increased mortality (5.6%), length of stay (2.2 days), rates of colectomy (3.5%), and hospitalization costs ($26,183) when compared to IC alone. Based on these findings, increasing the clinical suspicion and close observation of IC in patients with CHF who present with bleeding per rectum might be warranted. Furthermore, outcomes may vary with the degree of heart failure, anatomic area of colonic involvement, and presence of other comorbidities not included in our study, and we recommend further research in these areas regarding this topic. In addition, we advocate for exploration into race and gender as possible prognostic indicators for IC. Finally, hospitalization costs are significantly higher in patients with IC and CHF, and we further recommend that future studies focus on costs associated with comorbidities in the setting of IC to facilitate the development of healthcare policy.
